# Effect of Far-Red Light and Nutrient Solution Formulas on Calendula Production in a Plant Factory

**DOI:** 10.3390/biology14060716

**Published:** 2025-06-18

**Authors:** Maitree Munyanont, Na Lu, Thanit Ruangsangaram, Michiko Takagaki

**Affiliations:** 1 Graduate School of Horticulture, Chiba University, 648 Matsudo, Matsudo 271-8510, Chiba, Japan; maitree_m@tistr.or.th (M.M.); rsar.thanit@gmail.com (T.R.); 2 Lamtakhong Research Station, Expert Center of Innovative Agriculture, Thailand Institute of Scientific and Technological Research, Khlong Luang 12120, Pathum Thani, Thailand; 3 Center for Environment, Health and Field Sciences, Chiba University, 6-2-1 Kashiwanoha, Kashiwa 277-0882, Chiba, Japan; mtgaki@faculty.chiba-u.jp

**Keywords:** edible flower, light spectrum, end-of-day far-red, NH_4_^+^, nutrient use efficiency

## Abstract

Calendula (*Calendula officinalis* L.), an edible and medicinal flower, faces challenges in yield uniformity and quality stability under open-field cultivation. Plant factories with artificial lighting (PFALs) are highly controlled systems that enable year-round production, efficient resource use, and consistent crop quality, making them promising for the cultivation for calendula. To optimize calendula cultivation in PFALs, this study investigates the effects of far-red (FR) light and nutrient solution modification on calendula growth, flowering, and nutrient use efficiency (NUE). The experiment was conducted using a dwarf calendula cultivar, ‘Orange Gem’. After transplanting, seedlings were subjected to a 2 × 3 factorial design comprising white (W) and end-of-day far-red (EOD-FR) lighting, in combination with three ammonium (NH_4_^+^) levels (1/3×, 1×, and 3× of the Enshi formula). The EOD-FR lighting and the increase in the NH_4_^+^ level resulted in taller plants and earlier first flower appearance. The shoot fresh and dry weight, the number of flowers, and the flower yield also increased under the higher NH_4_^+^ concentration, regardless of the light treatment. The 3× NH_4_^+^ treatment tended to enhance the nutrient absorption, leading to the highest nutrient use efficiency (NUE) based on the flower yield. These findings suggest that the application of EOD-FR lighting and high NH_4_^+^ in nutrient solution can improve calendula productivity and resource-use efficiency in PFALs.

## Simple Summary

Calendula, a valuable edible and medicinal flower, often shows uneven yield and quality in open fields. Plant factories with artificial lighting (PFALs) offer year-round high-yield production and consistent quality of plants, making them a promising method for calendula cultivation. This study investigated how far-red (FR) light and ammonium (NH_4_^+^) levels affect calendula growth and nutrient use efficiency. Using the ‘Orange Gem’ cultivar, plants were grown under two light treatments (white and end-of-day FR) and three NH_4_^+^ levels (1/3×, 1×, 3× Enshi formula). Results showed that higher NH_4_^+^ increased plant height, flower number, and yield. End-of-day FR light also promoted earlier flowering. The 3× NH_4_^+^ treatment led to the highest nutrient use efficiency. These results suggest that EOD-FR lighting, combined with high NH_4_^+^ levels, can enhance calendula productivity in PFALs.

## 1. Introduction

*Calendula officinalis* L. (commonly known as calendula or pot marigold) is receiving increased attention due to its edible and medicinal properties. Calendula has been used traditionally in local dishes such as salads and soups for its vibrant color and mild peppery taste [1]. It is also widely used in herbal tea products, valued for both its esthetic and health-promoting qualities [2]. The plant contains a rich profile of bioactive compounds, including phenolics, flavonoids, carotenoids, and essential oils, known for their anti-inflammatory, antimicrobial, and antioxidant properties [3]. However, conventional open-field cultivation of calendula often results in seasonal yield fluctuations, product non-uniformity, and an inconsistent phytochemical composition [4].

Plant factories with artificial lighting (PFALs) are advanced cultivation technologies for controlled-environment agriculture that allow for precise regulation of environmental conditions, including light, temperature, humidity, and nutrient supply. Unlike traditional open-field or greenhouse cultivation, PFALs significantly reduce environmental fluctuations, thereby improving yield consistency and resource-use efficiency (RUE), and enabling year-round production [5,6]. Various crops have been successfully cultivated in PFALs, such as leafy vegetables (lettuce [7], basil [8], and coriander [9]), small-sized fruit crops (tomato [10,11], strawberry [12]), and medicinal plants [13]. These highlight the potential of PFALs to optimize calendula production under stable, controlled conditions to improve the yield and quality.

Light is one of the most critical environmental factors in PFALs, as it directly regulates plant photosynthesis, morphogenesis, and flowering [14]. Among light properties, the spectrum—particularly the presence of far-red (FR) light—plays an important role in plant development. FR light (700–750 nm) interacts with plant phytochromes, which exist in two forms: the active form (Pfr, absorbing red light of ~660 nm) and the inactive form (Pr, absorbing FR light of ~730 nm) [15]. Exposure to FR light shifts the balance toward the Pr form, leading to shade avoidance responses such as stem elongation, leaf expansion, and in some species, earlier flowering. Numerous studies have reported that FR light enhances flowering across various ornamental species. For instance, night-break FR treatments promoted early flowering in *Eustoma grandiflorum*, despite an increase in internode length [16]. Similar trends were observed in petunia and snapdragon under low daily light integral conditions, where FR light addition accelerated flowering [17], and EOD-FR light shortened the time to flowering in species like *V. tricolor*, *G. muralis*, and *D. chinensis* [18]. Additionally, FR treatments have been shown to enhance biomass accumulation, as observed in lettuce, where dry weight increased by 46–77% depending on plant density [19] and by 36% under daytime FR light and 94% under the EOD-FR treatments [20]. Despite these findings, plant responses to FR light vary based on cultivation conditions and application methods.

Hydroponic cultivation systems are commonly used in PFALs and the nutrient solution is one of the key factors for plant growth and development. A nutrient solution provides all essential nutrients required for plant growth, containing a combination of macronutrients and micronutrients dissolved in precise concentrations to support plant growth in soilless culture. Among the essential nutrients, nitrogen (N) is critical in plant metabolic processes [21]. The effects of N on plant growth have been widely studied. It is reported that N enhances leaf area and increases shoot biomass by increasing chlorophyll content and CO_2_ assimilation rate. N also promotes root development, improving nutrient uptake, and contributes to maintaining nutrient balance [22]. Additionally, nitrogen availability has been shown to influence flowering regulation [23,24]. A U-shaped response of flowering to N concentration was reported, and the optimal N level that leads to early flowering was found. Being above or below the threshold N level results in delayed flowering [25]. Plants absorb N mainly in the form of nitrate (NO_3_^−^) and ammonium (NH_4_^+^), and the preferred form depends on the species and environmental conditions [26]. Notably, NO_3_^−^ accumulation in edible tissues poses health concerns, making NH_4_^+^ a more suitable N source in some cases.

Although lighting strategies and nutrient formulations have been extensively studied, the combination of FR and nutrient solution composition remains underexplored, particularly within the context of PFAL cultivation. Adjusting both light spectrum and nutrient regimes has the potential to maximize plant productivity by improving plant growth, flowering, and resource-use efficiency (RUE). Therefore, this study aims to investigate the effects of FR light and nutrient solution modifications on calendula cultivation in a PFAL, specifically focusing on the effects of EOD-FR light in combination with NH_4_^+^ concentrations on the flower production and nutrient use efficiency. The outcomes are expected to provide practical insights for improving edible flower production in PFALs, using calendula as a model crop species.

## 2. Materials and Methods

### 2.1. Plant Cultivation

Efficient space use in PFAL cultivation is essential. Therefore, the dwarf calendula cultivar ‘Orange Gem’ (Takii & Co., Ltd., Kyoto, Japan) was grown for this study. Seeds were sown in a rock wool cube (5 cm × 5 cm × 5 cm), and they were kept in the dark at 15 °C. After 72 h, germinated seedlings were transferred to the light for a 12 h photoperiod and a light intensity of 240 ± 10 µmol m^−2^ s^−1^. The temperature was maintained at 23 ± 2 °C for the whole day. Enshi nutrient solution was prepared with an electrical conductivity (EC) and pH of 1.2 ± 0.1 dS m^−1^ and 6.5 ± 0.1, respectively. Nutrient solution was supplied hydroponically for 15 min per day through the irrigation system for 21 days until transplanting. Then, the uniform seedlings with four true leaves were transplanted to the treatments using a deep-water culture hydroponic cultivation system. This experiment was conducted with the combination of two lightings, W and EOD-FR light, and three NH_4_^+^ concentrations, 1/3 times (1/3×), 1 time (1×), and 3 times (3×) the Enshi’s nutrient solution formula (Table 1). W light was set at 12 h of photoperiod with 300 ± 10 µmol m^−2^ s^−1^ of light intensity (DLI = 12.96 mol s^−2^ day^−1^), and the EOD-FR treatment using the same W setting was added with 80 ± 5 µmol m^−2^ s^−1^ of FR for 6 h after the end of the W lighting period. As the NH_4_^+^ treatments, the nutrient solutions were prepared in a 100-times stock solution (modification recipes are shown in Table S1). The residual nutrient solution was kept, and the stock solution was added in 7.5 mL per liter every 10 days in the vegetative stage and 14 days in the reproductive stage. During the experiment, the water level was maintained at a sufficient volume for plant growth by adding 1 L every two or three days. Temperature, humidity, and CO_2_ concentration were maintained at 23 ± 2 °C, 50–70%, and 1000 ± 50 ppm, respectively.

### 2.2. Measurements and Statistical Analysis

The growth parameters, i.e., plant height (from the surface of the pot to the top of the main stem at the day of first flower bud appearance), shoot fresh weight (stem, leaves, and flowers were weighed separately, then summed to obtain a shoot fresh weight), and the days from sowing to the appearance of the first flower bud, were observed directly from the plants. Flower yield was observed by counting the number of flowers and computing the flower fresh and dry yield by weight at 104 DAS. Shoot samples were placed into a hot-air oven at 80 °C for 3 days for dry weight after checking the fresh weight. The leaf area was analyzed using image processing software (ImageJ version 1.54g, National Institutes of Health, MD, USA). Leaf mass per area (LMA) was calculated by the ratio of leaf dry weight to leaf area. For the photosynthetic pigment measurement, the exacted weight of fresh leaf samples was immersed in 2 mL of N, N-dimethylformamide (DMF), and kept under dark conditions for 36 h. Then, the aliquot was measured for the absorbance of the extracts at 480, 645, and 663 nm using a spectrophotometer (SH−1300 Lab, Corona Electric Co., Ltd., Hitachinaka, Ibaraki, Japan). Pigment concentrations were calculated using the following equations, and the value was shown in milligrams per gram of fresh weight (mg g^−1^ FW). [27,28]:

(1) Chl A=11.65A664−2.69A647

(2) Chl B=20.81A664−4.53A647

(3) Total chlorophyll content=Chl A+Chl B

The values of EC, pH, and nutrient ions were tracked during the cultivation period. An ion chromatography system (ICS-1100, Thermo Fisher Scientific, Inc., Tokyo, Japan) was used to analyze the ions in the nutrient solution. The nutrient absorption (NA), nutrient absorption efficiency (NAE), nutrient waste (NW), and nutrient use efficiency (NUE) were calculated as follows [8]:

*NA = total amount of applied nutrients (TAN)—the final amount of nutrients remaining (FAN)* (4)

*NAE = NA/TAN* (5)

*NW = FAN/Plant dry weight* (6)

*NUE = Flower yield/NA* (7)

For statistical analysis, the two-way ANOVA method was performed to investigate the interaction between the two factors described by Wei et al. [29]. Tukey’s post hoc test was used for mean comparison using SPSS statistical analysis software (IBM SPSS Statistics, Version 19.0, Armonk, NY, USA: IBM Corp.). The results were considered as significant differences when a *p*-value < 0.05.

## 3. Results

### 3.1. Growth Parameter

**Figure 1:**
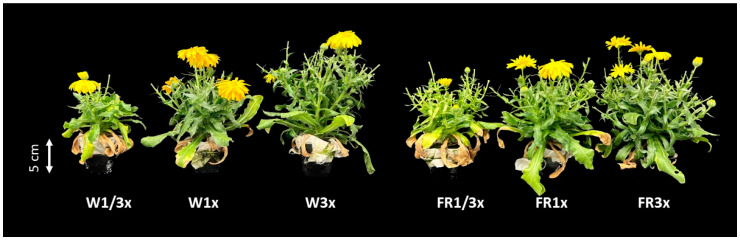
Morphology of calendula cultivated in PFALs with light and NH_4_^+^ at 104 DAS.

**Figure 2:**
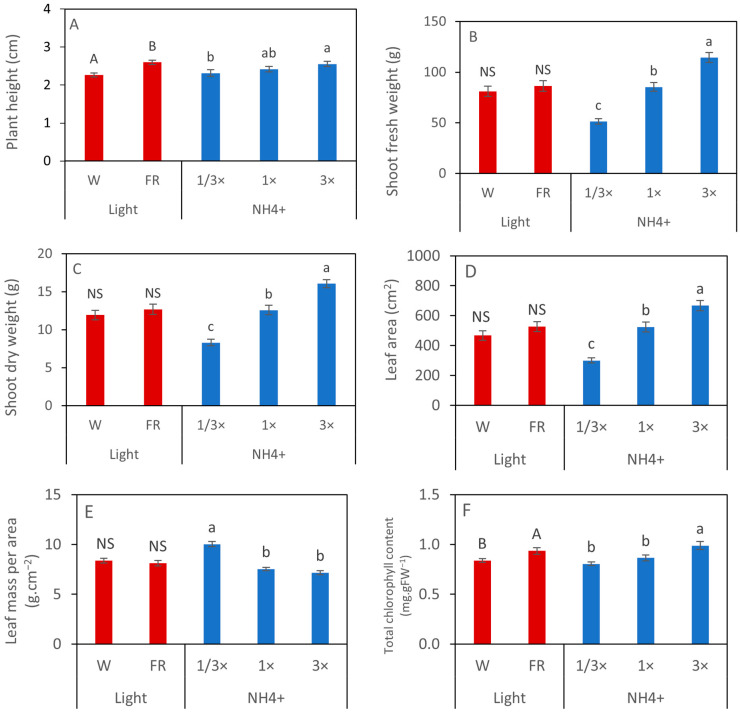
Plant height (**A**), shoot fresh and dry weight (**B**,**C**), leaf area (**D**), leaf mass per area (**E**), and total chlorophyll content (**F**) of calendula cultivated in PFALs with different combinations of light and NH_4_^+^. Data are shown in mean ± SE (*n* = 45 for light treatment and *n* = 30 for NH_4_^+^ treatments). Different letters indicate significant differences at *p* < 0.05 according to Tukey’s test, while ‘ns’ denotes no significant difference. The capital letters show the differences in light treatments, and the small letters show the differences within the NH_4_^+^ treatments.

The morphological characteristics and growth responses of calendula cultivated under different lighting and nutrient solution conditions in a PFAL demonstrated significant variations. Because the interaction between light spectra and NH_4_^+^ concentration factors was not found, the results are presented by the main effect of each factor. Calendula plants exhibited distinct growth in response to different light and NH_4_^+^ treatments with variations in appearance and morphology (Figure 1). The taller plant under the EOD-FR treatment indicated that FR light more significantly influenced stem elongation than the W light. Meanwhile, increasing the NH_4_^+^ concentration increased plant height. Plants cultivated under 3× NH_4_^+^ exhibited greater height than those at 1/3× NH_4_^+^. However, there was no significant difference between 1× NH_4_^+^ and 3× NH_4_^+^ treatments (Figure 2A). The shoot weight was increased by the elevated NH_4_^+^ concentration, regardless of the FR effect. A similar response was observed in both parameters, shoot fresh and dry weight, and both parameters increased significantly with the increasing NH_4_^+^ concentrations. The highest shoot fresh and dry weight was observed under the 3× NH_4_^+^ treatment (Figure 2B,C).

The calendula leaf tended to be enlarged by the EOD-FR light; however, there was no significant difference in leaf area between W and EOD-FR treatment. The elevated NH_4_^+^ levels significantly increased the leaf area. Plants grown under 3× NH_4_^+^ exhibited the largest leaf area among all NH_4_^+^ concentrations (Figure 2D). In contrast, LMA showed an opposite trend. The EOD-FR lighting did not influence the LMA value, whereas the lowest NH_4_^+^ concentration (1/3× NH_4_^+^) resulted in plants having the highest LMA (Figure 2E). The total chlorophyll content in the leaf was analyzed, and W-treated plants showed a significantly lower level compared to other treatments. Additionally, the highest total chlorophyll content was observed under 3× NH_4_^+^, while there was no significant difference between 1/3× NH_4_^+^ and 1× NH_4_^+^ treatments (Figure 2F).

### 3.2. Flowering Parameters

**Figure 3:**
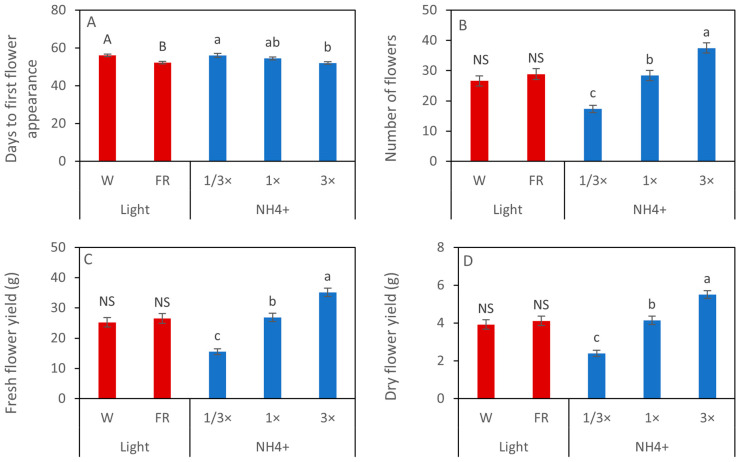
Days to first flower appearance (**A**), number of flowers (**B**), and fresh and dry flower yields (**C**,**D**) of calendula cultivated in PFALs with different combinations of light and NH_4_^+^. Data are shown in mean ± SE (*n* = 45 for light treatment and *n =* 30 for NH_4_^+^ treatments). Different letters indicate significant differences at *p* < 0.05 according to Tukey’s test, while ‘ns’ denotes no significant difference. The capital letters show the differences in light treatments, and the small letters show the differences within the NH_4_^+^ treatments.

Interestingly, EOD-FR-treated plants produced the first flower bud significantly earlier than W-treated plants. Similarly, increasing the NH_4_^+^ amount in the nutrient solution significantly accelerated the first flower bud appearance of calendula cultivated in PFALs. Plants grown under the lower NH_4_^+^ concentration (1/3× NH_4_^+^) took a longer time to display the first flower bud appearance than in the highest NH_4_^+^ concentration (3×) (Figure 3A). The number of flowers was not affected by the EOD-FR light, while it was significantly influenced by NH_4_^+^ level. The highest number of flowers was obtained from the plants grown under the 3× NH_4_^+^ concentration (Figure 3B). Furthermore, both fresh and dry flower yields followed a similar trend to the number of flowers. The light treatments did not significantly affect the flower yield; however, NH_4_^+^ concentrations showed a significant effect on flower yield. The highest fresh and dry flower yields were obtained under 3× NH_4_^+^, followed by 1× NH_4_^+^ and 1/3× NH_4_^+^, respectively (Figure 3C,D).

### 3.3. Change in Nutrient Solution

**Figure 4:**
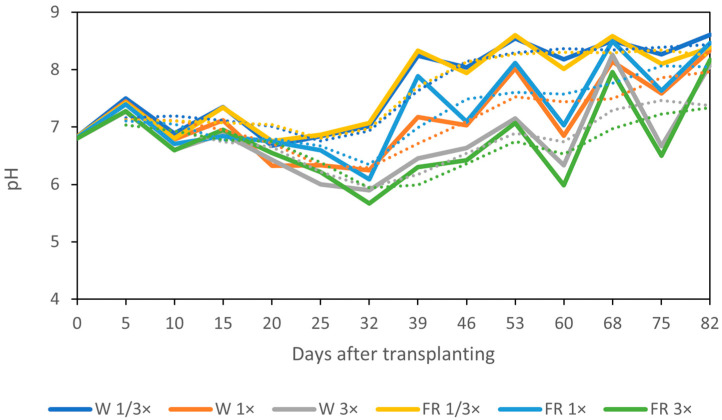
Changes in pH in nutrient solution under different light and NH_4_^+^ treatments. A dashed line shows the trend line of each treatment.

Figure 4 shows the variations in the pH in the nutrient solution throughout the cultivation period after transplanting. The initial pH was controlled at 6.8 ± 1 on the transplanting day for all treatments. The pH value fluctuated, with the rising pH occurring on the day before adding the nutrient solution. However, it tended to decrease gradually from the day after transplanting until day 32, then slightly increased until the last harvesting day (a dashed line). The NH_4_^+^ concentration was related to the pH regardless of the light treatments. When plants started to produce flowers (32 days after transplanting), the pH of each NH_4_^+^ treatment showed a different trend. The highest pH value was obtained from 1/3× NH_4_^+^ (8.3–8.5), followed by 1× (7.2–7.6) and 3× (6.5–7.0), respectively.

**Figure 5:**
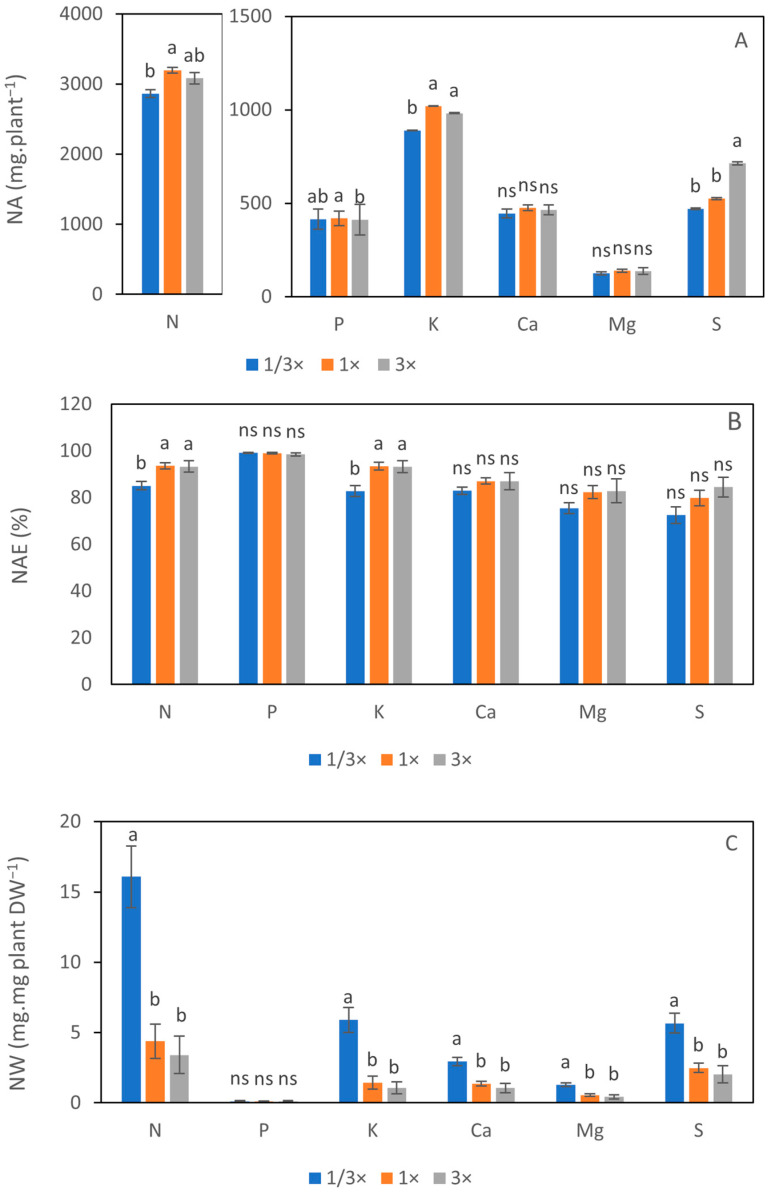
Nutrient absorption (NA) (**A**), nutrient absorption efficiency (NAE) (**B**), and nutrient waste (NW) (**C**) of N, P, K, Ca, Mg and S after 104 days of cultivation under different NH_4_^+^ treatments are presented. The error bars represent SEs (*n* = 3). Different letters indicate significant differences at *p* < 0.05 according to Tukey’s test, while ‘ns’ denotes no significant difference.

**Figure 6:**
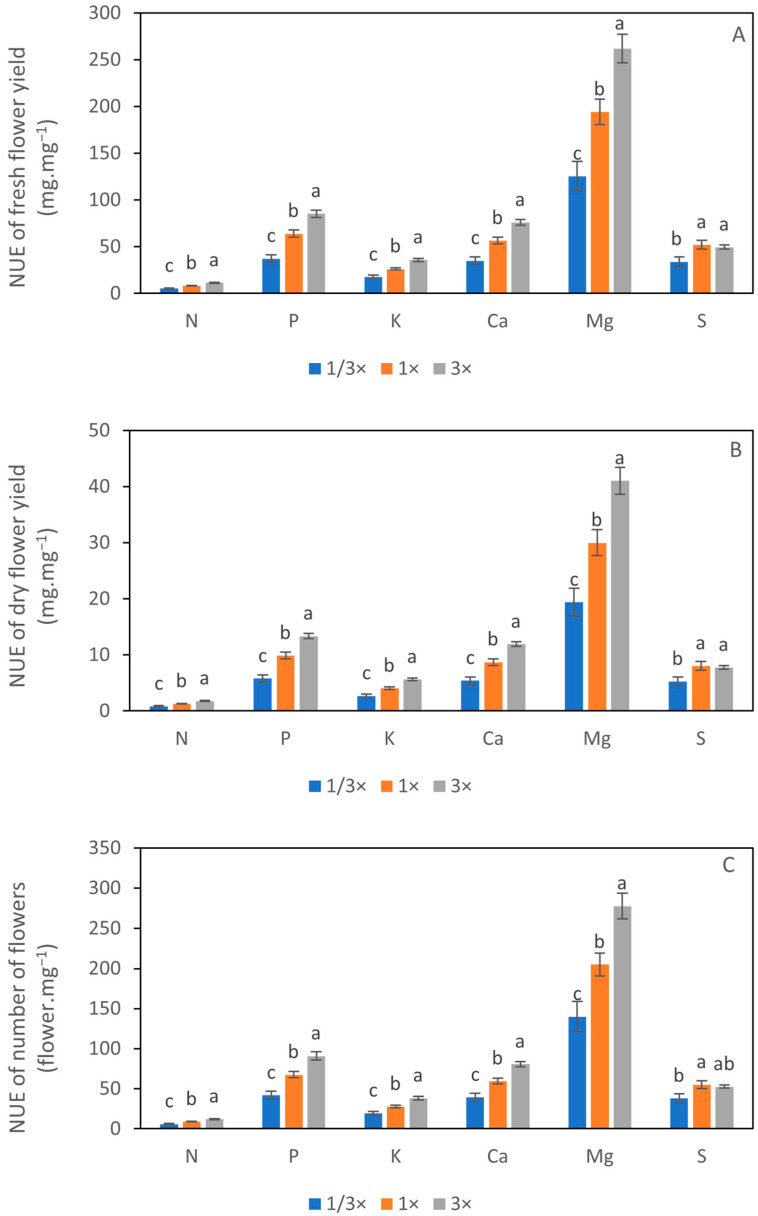
Nutrient use efficiency (NUE) of fresh (**A**) and dry flower yields (**B**), and the number of flowers (**C**) after 104 days of cultivation under different NH_4_^+^ treatments. Data showed the NUE of N, P, K, Ca, Mg, and S with SEs (*n* = 3). Different letters indicate significant differences at *p* < 0.05 according to Tukey’s test.

Nutrient absorption (NA) of N, P, K, and S was significantly influenced by NH_4_^+^ level. The N absorption of plants cultivated in the 1× NH_4_^+^ treatment was higher than that of 1/3× NH_4_^+^. However, it was not significantly different from 3× NH_4_^+^. The absorption of P under the 1× NH_4_^+^ treatment was higher than the 3× NH_4_^+^ treatment; however, there was no significant difference between 1× NH_4_^+^ and 1/3× NH_4_^+^. A similar trend to the N absorption was found in the K. The K absorption of 1× NH_4_^+^ and 3× NH_4_^+^ was higher than that of 1/3× NH_4_^+^. The highest absorption of S was observed under the 3× NH_4_^+^ treatment, while 1/3× NH_4_^+^ and 1× NH_4_^+^ showed insignificant differences. The absorption of Ca and S was similar under the different NH_4_^+^ treatments (Figure 5A). Comparing the nutrient absorption efficiency (NAE), the result demonstrates that the NAE remained consistently high across all treatments, above 80% in N, P, K, and Ca and over 70% in Mg and S. Interestingly, there was a significant difference in the NAE for N and K, showing a similar trend, with the lowest NAE in 1/3× NH_4_^+^. However, the NAE of P, Ca, Mg, and S did not show a statistical difference (Figure 5B). The nutrient waste (NW) of N, K, Ca, Mg, and S was the highest in 1/3× NH_4_^+^ among the NH_4_^+^ treatments, whereas the NW of P was unaffected (Figure 5C). The nutrient use efficiency (NUE) was presented under three scenarios, including fresh and dry flower yields and the number of flowers. The NUE was increased with the elevated NH_4_^+^ concentration regardless of the yield types. Plants grown under the 3× NH_4_^+^ exhibited the highest NUE of N, P, K, Ca, and Mg, while the NUE of S showed a non-significant difference between 1× NH_4_^+^ and 3× NH_4_^+^ (Figure 6A–C).

## 4. Discussion

### 4.1. Shade Responses and Biomass Accumulation Under FR Condition

This study confirmed that calendula exhibits shade-avoidance responses, such as stem elongation under FR light. The EOD-FR light application resulted in significantly taller plants than the W light treatment. This effect is consistent with previous reports showing that FR light promotes stem elongation by increasing the endogenous level of indole-3-acetic acid (IAA), a key auxin involved in cell division and elongation, particularly under a low red-to-far-red (R: FR) light ratio [30]. Although biomass accumulation and leaf area did not show a statistically significant difference, there was a trend indicating that plants grown under FR light tended to accumulate more biomass and a larger leaf area compared to those grown under W light. It is reported that FR light application not only altered morphology but also enhanced light capture. The increased leaf area under FR light may contribute to greater light interception, leading to improved light-use efficiency and cumulative dry matter production, as seen in lettuce and ornamental species [16,31,32].

### 4.2. Flowering Time Regulated by FR Light

Notably, our results demonstrated that EOD-FR light significantly accelerated the appearance of the first flower. Prior studies on marigold and petunia showed that earlier flowering was obtained under FR-enriched conditions [33,34]. This observation also aligns with findings in petunia that EOD-FR light reduced the time to flowering by 2–7 days [35,36]. Inversely, the delay of flower budding was observed under FR-deficient conditions, particularly in long-day plants [37,38]. The mechanism is likely tied to the activation of flowering-related genes such as CONSTANS (CO) and FLOWERING LOCUS T (FT) via phytochrome signaling, which responds distinctly to FR light at the end of the photoperiod [39,40]. Given that calendula is a facultative long-day species, these findings support the hypothesis that FR light manipulation—especially EOD-FR light—can be used strategically to regulate flowering timing in PFALs, providing better control over harvest scheduling.

### 4.3. Plant Growth, Flowering, and NUE Were Promoted by Elevated NH_4_^+^

This study focused on modifying NH_4_^+^ concentrations while maintaining constant EC levels. Increasing NH_4_^+^ levels significantly improved plant growth parameters such as plant height, dry weight, leaf area, and chlorophyll content as well as flower yield. The best performance was observed under the 3× NH_4_^+^ treatment, which employed an NH_4_^+^: NO_3_^−^ ratio of 20:80. This treatment also exhibited a higher nutrient absorption efficiency and nutrient use efficiency (NUE) across most macronutrients (Figure 5 and Figure 6). When the nutrient solution is supplied to the plants, the form of NH_4_^+^ and NO_3_^−^ can be altered to maintain the total N content constantly and may modify the total cation to anion absorption ratio. The change in the total cation-to-anion uptake ratio affects the pH of the nutrient solution, directly influencing the absorption of macro- and micronutrients from the solution [41]. Interestingly, this NH_4_^+^: NO_3_^−^ ratio closely resembles the 25:75 ratio reported to enhance growth in flowering Chinese cabbage, where pH was stabilized within the optimal range of 5.64–6.50 [42]. The similar mechanisms may have contributed to the improved performance in calendula in our study. Furthermore, the enhanced chlorophyll content and expanded leaf area observed under 3× NH_4_^+^ treatment may have increased the photosynthetic capacity through improved light interception, a pattern that aligns with previous studies on leafy vegetables [43]. In addition to supporting vegetative growth, elevated NH_4_^+^ levels also advanced flowering by accelerating the appearance of the first flower bud. This effect is consistent with findings in *Arabidopsis thaliana*, a facultative long-day plant, where the time of long-day-induced flowering was reduced when either NH_4_^+^ or NO_3_^−^ concentration increased. Moreover, higher NO_3_^−^ levels were associated with the upregulated expression of flowering-related genes, suggesting that the nitrogen status could regulate plant flowering [44]. It was expected that there would be a threshold in N concentration affecting flowering time [25]. However, a threshold was not determined in the present study, probably due to the difference in cultivar or other environmental factors. Further research needs to be conducted.

## 5. Conclusions

This study demonstrated that both EOD-FR light and a high NH_4_^+^ concentration can independently enhance calendula performance in PFALs. Although no interaction was observed between FR light and NH_4_^+^ treatments, each factor contributed distinctly to plant development and productivity. The EOD-FR light application stimulated shade-avoidance responses and accelerated flowering, offering a practical strategy to hasten harvesting in PFALs. The higher NH_4_^+^ concentrations enhanced vegetative growth, chlorophyll content, flower productivity, and nutrient-use efficiency (NUE) across multiple macronutrients. Collectively, these findings highlight the possible strategies for optimizing growth and resource use efficiency in calendula cultivated in PFALs. The absence of interaction between EOD-FR lighting and NH_4_^+^ concentrations indicates that these treatments can be applied independently without negative interaction effects. This flexibility allows growers to adopt either or both strategies according to production goals and resource availability. Such an approach offers a practical and adaptable cultivation strategy for edible flower producers aiming to enhance productivity and sustainability in PFALs.

## Figures and Tables

**Table 1:** The experimental design and treatments.

		Light Treatments			
		W		EOD-FR	
Intensity (µmol m^−2^ s^−1^)	W	300 ± 10		300 ± 10	
	FR	-		80 ± 5	
Photoperiod (h)	W	12 (6:00–18:00)		12 (6:00–18:00)	
	FR	-		6 (18:00–0:00)	
		NH_4_^+^ Treatments			
		1/3×	1×		3×
NH_4_^+^ concentration (me/L)		0.43	1.3		3.9
NH_4_^+^:NO_3_^−^ ratio		3:97	7.5:92.5		20:80
EC (dS m^−1^)		1.8	1.8		1.9

## Data Availability

The original contributions presented in this study are included in the article and Supplementary Materials. Further inquiries can be directed to the corresponding author.

## Supplementary Materials

The following supporting information can be downloaded at https://www.mdpi.com/article/10.3390/biology14060716/s1: Table S1: Enshi nutrient solution formula and the NH_4_^+^ modification for treatments.
