# Supplementary material for: Effect of Far-Red Light and Nutrient Solution Formulas on Calendula Production in a Plant Factory
Source: Biology (Basel). 2025 Jun 18;14(6):716. doi: 10.3390/biology14060716 (PMC12189887; doi:10.3390/biology14060716)
Supplement: Supplementary file 1 [file biology-14-00716-s001.zip › biology-3688668-supplementary.pdf]

Table S1 Enshi nutrient solution formula and the NH_4^+ modification for treatments

	1/3x	1x (standard Enshi recipe)	3x
NH_4^+ (me/L)	0.43	1.3	3.9
NO_3^- (me/L)	16	16	16
NH_4^+:NO_3^- ratio	2.6: 97.4	7.5: 92.5	19.6: 80.4
100x stock solution (g L^{-1})			
Stock A			
Ca $(NO_3)_2.4H_2O$	94.5	94.5	94.5
Stock B			
KNO_3	80.9	80.9	80.9
$NH_4H_2PO_4$	4.9	15.3	15.3
$MgSO_4.7H_2O$	49.3	49.3	49.3
NaH_2PO_4	10.8	0	0
NH_4Cl	0	0	7.0
$(NH_4)_2SO_4$	0	0	8.6
Ion in 1x nutrient solution (mg L^{-1})			
NO_3^-	992.06	992.06	992.06
NH_4^+	7.76	24.05	70.96
PO_4^{3-}	126.31	126.63	126.63
K^+	312.78	312.78	312.78
Ca^{2+}	160.31	160.31	160.31
Mg^{2+}	48.60	48.60	48.60
SO_4^{2-}	192.10	192.10	254.54
